# Compounds with Epoxy Resins and Phase Change Materials for Storage in Solar Applications

**DOI:** 10.3390/ma12213522

**Published:** 2019-10-27

**Authors:** Miguel Ángel Álvarez Feijoo, María Elena Arce Fariña, Andrés Suárez-García, David González-Peña, Montserrat Díez-Mediavilla

**Affiliations:** 1 Defense University Center, 36920 Marín, Spainandres.suarez@cud.uvigo.es (A.S.-G.); 2 University of Burgos, 09006 Burgos, Spain; davidgp@ubu.es (D.G.-P.); mdmr@ubu.es (M.D.-M.)

**Keywords:** PCM, phase change material, Plackett-Burman, energy plus

## Abstract

Composite materials have great potential for growth due to their excellent properties and their multiple applications. The study of the thermal properties of the new composites resulting from the combination of epoxy resin and phase change materials (PCM), as well as thickening agents and thermally conductive compounds, was the objective of this work. For this purpose, different samples were manufactured by combining epoxy resins, organic PCMs (paraffins), and aluminum particles. Several properties were analyzed: thermal behavior (by differential scanning calorimetry technique), hardness, etc. To carry out this analysis, parameters of PCM quantity and metallic particles in the composition were varied. The results showed that the epoxy resin acted as a matrix containing the rest of the components and encapsulating the PCM. The organic PCM showed reversibility when subjected to multiple cycles. The enthalpy of the organic PCM–resin compound varied linearly according to the PCM content in the sample. For the content of this material in the samples to reach up to 40%, the use of thickening agents was necessary. The use of metallic particles improved the conductivity of the composites even while maintaining a low percentage by weight of metallic particles. Thermal simulations of the composite in bottom-coating a photovoltaic panel estimated a reduction of several degrees Celsius, showing the potential use of the PCM–epoxy resin for improving the energy production of such panels.

## 1. Introduction

The composite industry has very good growth prospects in the short and medium term due to the discovery of multiple applications for these materials. Their ability to combine with other substances of very different natures have led this type of material to be introduced in all areas of engineering, particularly in the solar sector as coatings. The composites have a number of advantages over traditional materials: weight reduction, simple and low maintenance, and better corrosion resistance are the key factors needed to maximize the useful life of any element in a solar thermal or photovoltaic installation [1].

Corrosion influences wear, malfunction, and breakage failures due to thermal and mechanical fatigue phenomena, causing a decrease in performance. Ultimately, the combination of these negative factors is reflected in a decrease in the efficiency of material and personal resources, as well as a detriment to the materials’ life cycle. Composites allow improved efficiency and reduced corrosion of the equipment or system. In today’s context, society demands a more effective use of energy as environmental awareness grows. Solar systems could become more energy efficient with the application of customized protectors. The incorporation of these compounds into certain systems, and the appropriate combination of phase change materials (PCM) with the rest of the cold generation system, would make it possible to reduce operating costs and the CO₂ emitted into the atmosphere. In addition, in many cases, significant energy savings and a reduction in polluting emissions can also be achieved [2]. The use of this type of compound can also cushion undesirable effects, such as performance losses in equipment due to overheating, since the incorporation of phase change materials into an epoxy resin allows the thermal conductivity of the composite to be improved [1].

The use of thermal energy to change phase is the most important feature of any PCM. They have a high fusion heat and are able to store or release large amounts of energy, especially in the form of latent heat [3,4]. Another very important feature of these materials is their ability to adapt to periods of energy demand, supplying energy when it is needed [5]. This type of substance also has excellent heat absorption and transfer capacities associated with phase change processes (melting and/or solidification), known as latent heat [6]. Materials that store energy in the form of latent heat are ideal for applications where there is a high energy density, where volume and weight are limiting in the system, or where the stored energy needs to be obtained at a constant temperature [7].

The most commonly used phase change materials are those that take advantage of a solid–liquid state change, and vice versa, due to the high energy absorbed or transferred and the reduced volumetric changes. A substantial variation in volume during the change of state is reflected in the appearance of internal stresses in the matrix containing the PCM and, therefore, a decrease in mechanical properties. Another advantage of these materials is that in the state-change process, the temperature variation is very low [8]. The main requirements for these substances are: melting and freezing temperatures in the range of the application, high latent heat of melting, and high thermal conductivity. The PCMs are classified into organic, inorganic, and eutectic categories. Organic PCMs include paraffin and non-paraffinic compounds (fatty acids, esters, alcohols, glycols, etc.). Inorganic PCMs are classified into hydrated salts and metallic materials. A eutectic is a minimum melting composition of two or more components (both organic and inorganic) that has a lower melting point than each component. Paraffin waxes are among the most commonly used materials for energy storage applications [9,10,11]. However, one of the main disadvantages of PCMs that display solid–liquid change is the possible loss of material when liquefying. This reduces heat transfer efficiency and increases production costs. To solve this problem, different solutions have been developed: microencapsulation, developed extensively in many lines of research, consists of coating the PCM with a layer of another material that interacts neither with the matrix nor with the PCM [12,13]; other lines of research have resorted to introducing the PCM directly into a matrix of another material that acts as a container material for the PCM [14]. Another disadvantage observed in organic PCMs is their low thermal conductivity, which significantly affects energy loading and unloading rates.

Photovoltaic (PV) systems convert solar energy into electricity. This conversion process is carried out by solar cells, which currently have an energy conversion efficiency of around 15%–20% [15]. The rest of the energy is converted into heat, which causes heating of the cells, reducing their efficiency [16]. The efficiency of the conversion of solar energy into electricity decreases for each degree that the temperature of the panel increases [17,18]. One of the most commonly used options to improve this efficiency is to cool the panel. The cooling of a photovoltaic panel can be active or passive. Active cooling systems use a liquid or gas for cooling. For heat extraction, fans, water recirculation pumps, etc. are needed. The variety of passive cooling systems is very wide. The simpler ones use fins or other surfaces, or incorporate metals with high thermal conductivity to increase the transfer of heat to the environment. The most complex systems use a PCM [15]. In systems that use a phase change material, the dissipation of heat to the environment is limited by the heat exchange surface between the panel and the environment, in which the convective heat transfer coefficient and the radiative heat transfer are determining factors [19]. With this type of cooling, no additional cooling is required in the PV panels. The ideal PCM will be the one with a high latent heat of fusion and high thermal conductivity, and that is chemically stable, non-corrosive, and non-toxic, with a low subcooling and state change temperature around the working temperature of the panel [20]. 

In this work, the use of epoxy resin as a polymer matrix was chosen to support the dispersed material, a phase change material in this case, giving a PCM–epoxy composite. Epoxy resins are considered high-performance substances, as their physicochemical properties are ideal. Their high adhesion power to other materials, especially metals (for example, the materials on the external surfaces of generators and exhaust pipes are metallic), makes them ideal as a coating. In order to reduce the temperature of photovoltaic panels, the possibility of incorporating a layer of resin with PCM onto the interior surface of the panel was studied. In this first approach to the problem, the objective was to detect the most relevant factors in its elaboration, using the design of the Plackett-Burman experiments and considering the different components used in its elaboration. At the time of defining relevance, a thermal analysis was carried out in order to weigh the thermal capacities and stability of the composite material. Although computational fluid dynamics (CFD) models have been widely used for natural ventilation simulations [21,22], they become unstable for conjugate heat transfer model analysis—that is the transient heat transfer between solid and fluid, as examined in this study. Thus, the PCM implementation into the solar panel was analyzed using a nodal model.

## 2. Materials and Methods 

**Figure 1:**
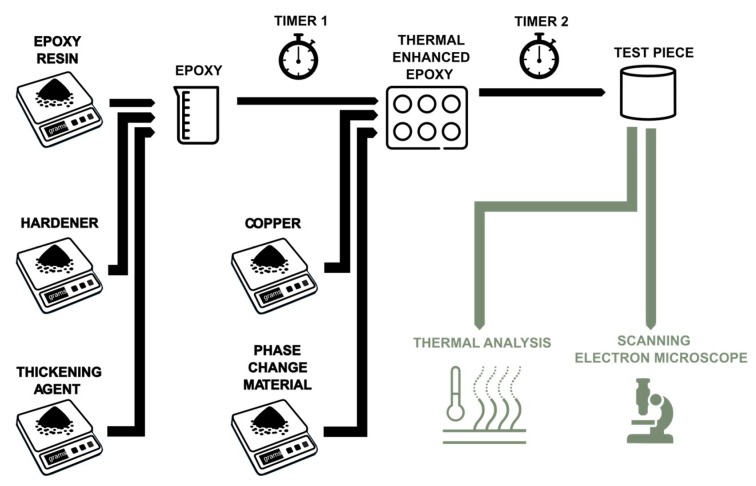
Workflow of the creation and testing of the phase change material (PCM)-epoxy samples.

The methodology was divided into three clearly different stages, which are the part of creating the PCM–epoxy test probes and their subsequent analysis (Figure 1) and simulation. For the production of samples, the different components were first weighed separately and then added at two times: during mixing, where the resin increases its viscosity as it passes; and during curing, where the specimen was left to rest before submitting it to laboratory tests. Both thermal and visual aspects were analyzed in these tests; in other words, the ability of the composite to absorb and release heat was characterized and the homogeneity of the sample was observed.

**Figure 2:**
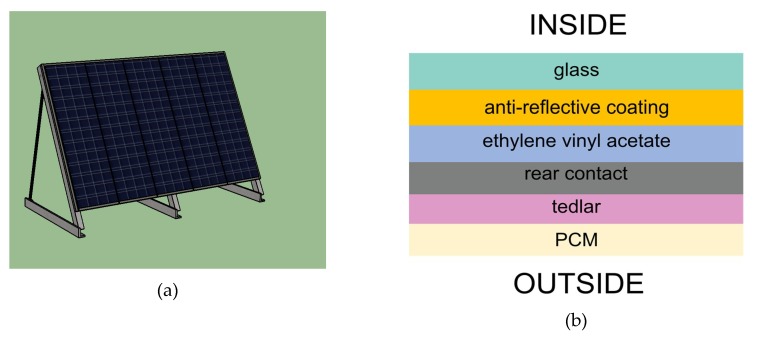
(**a**) Photovoltaic panel modeled in Sketchup; (**b**) panel configuration to see the position of the Phase Change Material (PCM).

The simulation for estimating the performance of the PCM consisted of two different solar panel configurations, with horizontal and location latitude inclination, in Burgos and Almería. The locations were selected based on their climate conditions. EnergyPlus [23] and TRNSYS [24] are common software suites utilized for modeling systems in order to corroborate experimental findings. Due to EnergyPlus’ capabilities, it was chosen for the present study. The conditions of the simulation were defined in EnergyPlus (v9.1). The geometric model used in EnergyPlus was made in SketchUp. Since the objective of the inclusion of the PCM was to dampen the panel temperature, only the temperature variable was evaluated. The Cp curves, thermal conductivities, densities, transition temperatures, and latent heats values generated experimentally were employed for the EnergyPlus modeling and simulation. The solar panel configuration was defined based on the research of Armstrong et al. [25]. Both panels (horizontal and inclined) were raised 0.5 m from the ground. Figure 2 shows the solar panel layer design included in EnergyPlus. The configuration of the layers of the panel did not allow more alternatives than the defined one. However, if such a PCM were applied in other solutions, such as building envelopes, it should be selected based on tested methodologies [26]. Because the solar panel included a PCM composite layer with variable thermal conductivity, the conduction finite difference (CondFD) solution algorithm was employed as a heat balance algorithm. The CondFD algorithm applies an implicit finite difference scheme with an enthalpy–temperature function to account the PCM energy. For the Burgos simulation, the panel was tilted at 42.35 °C and for Almería it was tilted 36.84 °C. These quantities were chosen to correspond to the respective geographic latitudes. The panel orientation was south in all cases. 

### 2.1. Components

In order to analyze the influence of the different components on the composite, several samples were prepared, varying the number of components in the mixture. For this purpose, the materials described in the following section were used.

(1) To carry out the study, the epoxy resin chosen was Resoltech 1070 ECO (with a density at 23 °C of 1.18 g/cm^3^ and a viscosity at 23 °C of 1750 mPa·s) in combination with a Resoltech 1074 ECO hardener (density at 23 °C of 0.96 g/cm^3^ and viscosity at 23 °C of 50 mPa·s), supplied by Castro Composites (Pontevedra, Spain). This resin has excellent wetting and adhesion properties, good electrical insulation, high mechanical and chemical resistance, and low solidification shrinkage. The composite obtained was transparent (allowing the encapsulation of all the substances that made up the matrix to be appreciated), with a glass transition temperature of around 73 °C.
(2) The PCM used was an organic material (paraffin), a wax called “candelilla”. This material was chosen among other paraffins for his outstanding adhesive, protective properties together with high chemical stability, impermeability, and dielectric properties. Like carnauba wax, it is highly recognized for its high hardness with wear resistance, and also acts as a moisture barrier due to its high hydrocarbon content. Due to the disadvantage of its low conductivity, it was combined with a powdered metallic material to increase thermal inertia. This paraffin has a melting range between 69 and 73 °C, which suited this application, an acidity index between 12 and 22 mg/g KOH, and a saponification index between 43 and 63 mg/g KOH. This wax, due to its melting temperature range, adapted better to the application than others.
(3) Garamite 1958 (Castro Composites, Porriño, Spain) was used as thickener. It is a substance easily mixed with the thermostable liquid base. The thickener is added to increase the thixotropy and viscosity of epoxy, polyester, and vinyl ester resins. It improves the no-hanging property of gels and resins in vertical walls. Its main function in this study was to boost the viscosity of the mixture, helping the mixture to be homogeneous and avoiding flocculation or suspension of particles due to different density.
(4) The metal particle added to the composite was powdered aluminum, because it does not require working temperatures as high as those of copper. This material was added with the aim of controlling the thermal inertia. The material properties indicate how much heat a substance can retain and whether the additive will contribute to rapid hardening of the substance. In addition, aluminum has a lower density than copper, a determining factor if a coating is desired to be used on vertical surfaces and must be as light as possible. Aluminum has a density of 2.7 mg/m^3^, its conductivity at 27 °C is 237 W/(m·K), its melting point is 660 °C, and its specific heat is 900 J/(kg·K). Graphite was discarded due to the poor surface finish of the specimen and its black tone, which results in a higher UV absorption than that of aluminum and copper.

### 2.2. Samples

The components were combined in the proportions defined below to prepare the different samples used in this study. First, the resin was well mixed with the hardener, and then the thickener was added (to avoid flocculation when the other components were added). Once the sample had been homogenized, PCM was added and mixed well. Finally, the metal particles were incorporated, stirring well, but avoiding the formation of air bubbles. The mixture was deposited in a silicone mold and left to solidify (approximately 24 h) before subsequent extraction. Cylindrical samples were obtained with a radius of 1.5 cm and a depth of 3 cm. It should be noted that the selected PCM came in solid particles too large to mix with the resin. When they were ground, a friction force was exerted between the mortar and the solids, generating a temperature variation. If the temperature reached was too high, the wax was liquefied. Therefore, an electric grinder was used to reduce particle size by grinding at low speed and setting aeration intervals to avoid the above problem.

The Placket–Burman method is a technique used to identify the most important factors involved in an experiment, which also allows the number of samples to be reduced. In this study, the Plackett–Burman experiment was designed considering five relevant factors (A, B, C, D, E) that were evaluated at two levels, +1 corresponds to larger proportions and −1 to smaller ones, as indicated in Table 1. The effect of several factors was analyzed, including the ratios of resin, catalyst, thickener, PCM, and metallic particles, and also the mixing time of the epoxy resin. The other two factors (F, G) used were dummy variables. These fictitious variables are used to distinguish factors with significant effect. That is, they can be used to estimate the effect of random errors, thus defining a minimum threshold of effect [15]. Any real variable with an effect is greater than this threshold can be considered statistically significant. In Table 2 there are defined each one of the factors in detail, also the values of their levels.

The total of all the mass factors added up to 20 g, which almost filled the volume of the specimen molds (which was 21.21 cm³). These molds were chosen because the dimensions of the specimens obtained were suitable for using them in compression tests if necessary. The ±1 level values were obtained by carrying out a series of previous tests for which the main purpose was to obtain a homogeneous sample without precipitates from any of the components (Table 2).

Beyond the mathematical model used to characterize the heat of fusion from the analyzed factors, the objective of using the Plackett–Burman design was to determine the statistically significant factors with the fewest experiments. Each one of the factor effects $E_{X}$ was compared against a critical value E_critical_. If the absolute value of $E_{X}$ was above the E_critical_, it can be said that the change in the factor had a significant impact on the response Y. The $E_{X}$ was determined as the mean of the responses of the dependent variable Y, the enthalpy in the present work, where the factor was at level + or – and N was the total number of experiments (1) [27].

(1) $$E_{X}=\frac{\sum^{\;}y_{+}-\sum^{\;}y_{-}}{N/2}$$

The E_critical_ was estimated using an ANOVA approach [28]. The one-way ANOVA is used to answer whether the variance between the means of two populations is significantly different. One population was made up of the real factors (i.e., A, B, C, D, E) and the other contained the dummy factors (i.e., F, G). For each factor, the sum of squares SS in a two-level design is given by Equation (2). The sum of squares of the real factors $SS_{X}$ can be compared against the means of the sum squares of the dummy $SS_{d}$ Equation (3). If $F_{X}$ is greater than one-tailed $F_{1,m}$ at the desired level of significance p, where m is the number of the number of dummy variables and p = 0.05 for this work, then the effect of changing the X is significant. In order to obtain the E_critical_, these steps have to be reversed, giving Equation (4).

(2) $$SS_{X}=\frac{N}{4}\;E_{X}^{2}$$

(3) $$F_{X}=\frac{SS_{X}}{SS_{d}}$$

(4) $$E_{critical}=F_{1,m}\;SS_{d}\;\frac{4}{N}$$

### 2.3. Analysis

Analysis of the thermal properties of the samples was carried out using a Thermogravimetry-Differential Scanning Calorimeter TG-DSC from the GTE group (Energy Technology Research Group) of the University of Vigo. This analysis involved two techniques that studied the change in behavior of the samples when they were subjected to a programmed temperature cycle under a controlled atmosphere. The heating–cooling cycle to which the samples were subjected is described below. A preliminary heating ramp at 5 °C/min was configured in the program to accelerate the heating process to 30 °C, and then this temperature was maintained for 2.5 min to stabilize the sample at that temperature. The next step in the cycle was to take the temperature to 80 °C with a heating ramp of 2 °C/min and, once reached, return to 30 °C again, cooling at 2 °C/min. At this temperature, samples were left 5 min to stabilize. These stages of heating, cooling, and stabilization were repeated another two times. Therefore, the sample was submitted to three cycles of temperature oscillation, from which the data of the last two were taken since the first one was discarded for stabilization.

An empty container, labeled "blank", in which the sample would later be placed for analysis, was submitted to a programmed cycle in the equipment, to study the thermal behavior of the container. The sample was then placed in the container and the heat flow curves of both the sample and the container were obtained, from which the results of the “blank” were subtracted to obtain the thermal behavior of the sample. 

The result of these measurements were the thermal analysis curves, and the characteristics of these curves (peaks, discontinuities, slope changes, etc.) are related to the temperature changes of the sample.

### 2.4. Sanning Electron Microscopy

Scanning electron microscopy (SEM) provided morphological information about the surface of the samples, generally necessary to understand their behavior and a very visual way of testing their homogeneity. X-ray microanalysis provided qualitative and quantitative information about the elements that made up the samples. The methodology was as follows:

(a) Preparation: the sample was fixed by adhesive to a 2.6 cm aluminum support and, to provide it with electrical conductivity, it was coated with by evaporation a thin layer of carbon.
(b) Observation: the analysis was carried out using a JEOL JSM6700F (JEOL, Munich, Germany) field emission scanning electron microscope, using a 15 kV beam. The equipment used belongs to the CACTI (Centre for Scientific–Technological Support to Research of the University of Vigo, Spain) [29].

## 3. Results and Discussion

**Figure 3:**
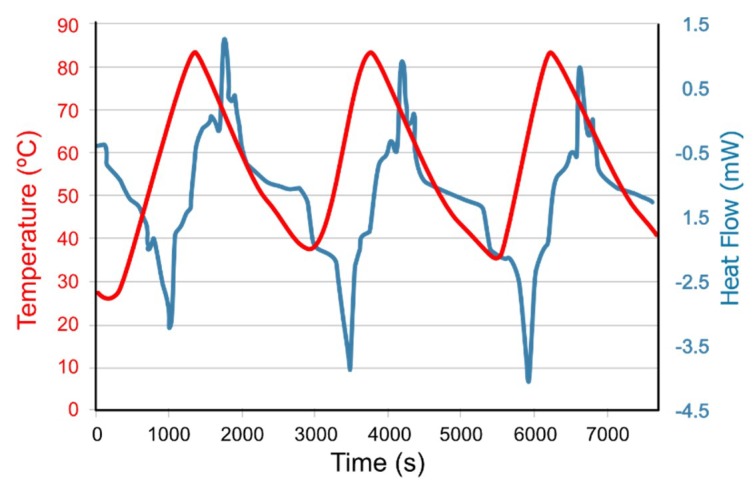
TG-DSC (Thermogravimetry-Differential Scanning Calorimeter) curves for sample 3.

The heat flow analysis in the TG-DSC equipment was used to study the effectiveness of mixing epoxy resin with PCM and aluminum. Peaks were defined to obtain the enthalpies (Figure 3). Of all the test samples analyzed, test sample 3 showed the highest peaks of heat absorption and release, i.e., the largest peak areas.

The obtained curves show that phase change transitions were quick, but not immediate, generating narrow, elongated, and differentiable peaks. In the solidification process, a small paraffin peak can be seen. It showed a scheme very similar to the curves generated by the PCM. This combination could be a good option for a coating, because it has a high heat absorption capacity accompanied by a subsequent thermal release, which allows the epoxy coating with thermal barrier effect to work under optimum conditions. Finally, a PCM sample recorded an enthalpy of 116.3203 J/g and −40.3127 J/g in the melting and solidification processes, respectively.

In the images obtained in the SEM-EDS, it was possible to observe the great homogeneity of the specimens (Figure 1). The matrix material was a mixture of epoxy resin and hardener, together with different solutes: thickener (mixture of different pure substances in solid state), candelilla wax or PCM (a mixture of different substances in solid state), and aluminum fillers (pure substance and in solid state). In general, the composition of the sample on the surface of the solid differred significantly from that of the interior of the solid. A quantitative analysis of microstructures of the order of 100 μm was carried out in order to identify the amounts of carbon, oxygen, aluminum, silicon, chlorine, and magnesium. In this analysis, the following weight percentages were determined: 75.32% C, 18.98% O, 2.26% Al, 0.18% S, 0.44% Si, 2.68% Cl, and 0.15% Mg.

**Figure 4:**
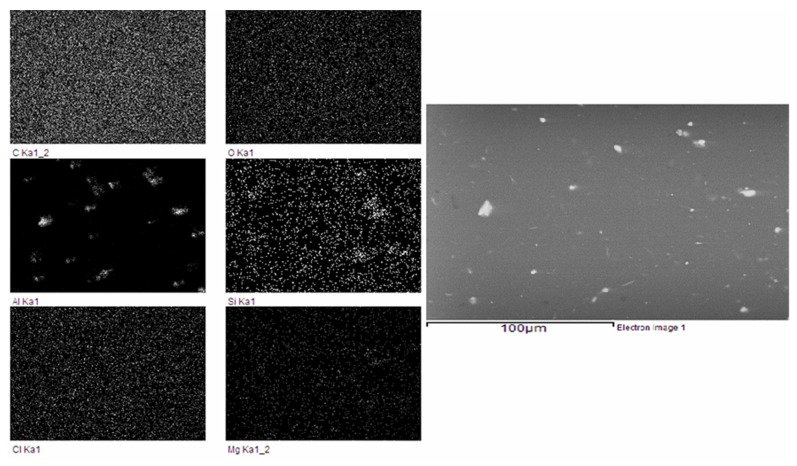
Mapping and sample composition for a 100 μm microstructure.

Figure 4 shows images of the different elements seen in the SEM-EDS. The element most clearly perceived was the aluminum, due to its greater atomic number, and the one that was least appreciable was the magnesium. Silicon was concentrated in small areas forming a brighter shade. In addition, sulfur appeared as an element to be taken into account, although its visualization in images was difficult due to its low atomic number. The sum of each of the components was used to generate the total image on the right. 

**Figure 5:**
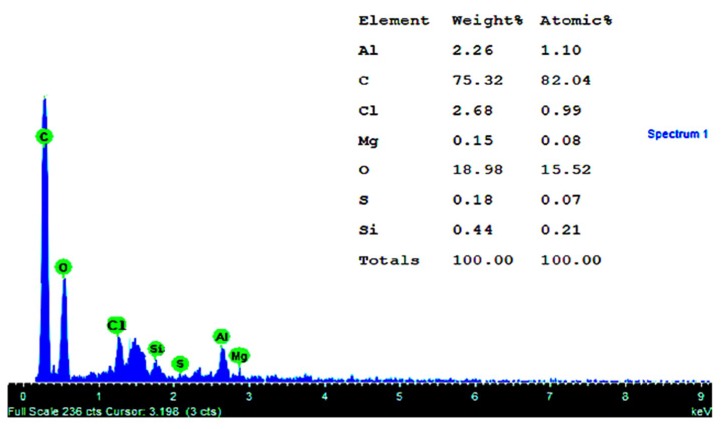
Atomic spectrum and quantification by scanning electron microscope and energy dispersive spectroscopy (SEM-EDS) equipment.

The comparative EDS results are shown in Figure 5, for both mass percent and percent atomic weight of the different chemical elements identified. EDS analyses showed an enrichment in carbon and oxygen (typical of candelilla wax and the epoxy group). The sulfur detected in the sample came from both the thickener and the resin hardener.

**Figure 6:**
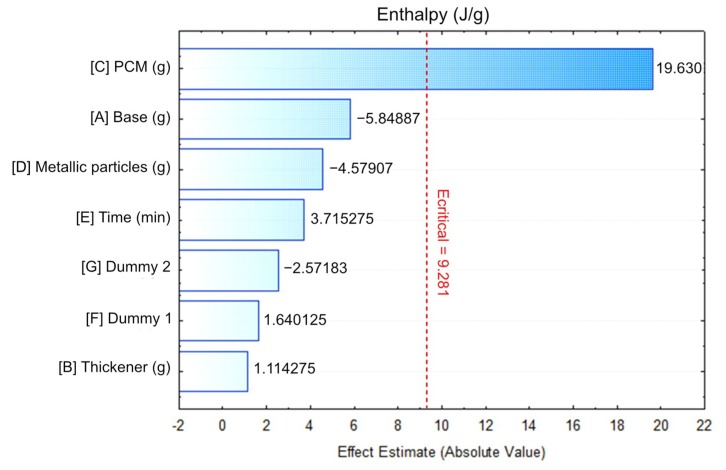
Pareto diagram of the Plackett–Burman experiment.

The Pareto diagram ranks, according to their importance, the factors that have contributed to the enthalpy of the different test tubes that made up the Plackett–Burman experiment (Figure 6). The graph consists of a group of bars that organizes the factors by descending order of influence on the dependent variables. The sign that accompanies the bars should be observed to identify whether the presence of the different factors was directly/inversely proportional to the thermal properties. The significance level of the effects was estimated at 9.281. Only one factor exceeded this figure. In other words, only the change of the PCM mass had a significant impact on the enthalpy. The other real factors were far from this critical value, indicating that their change was not relevant to the enthalpy.

**Figure 7:**
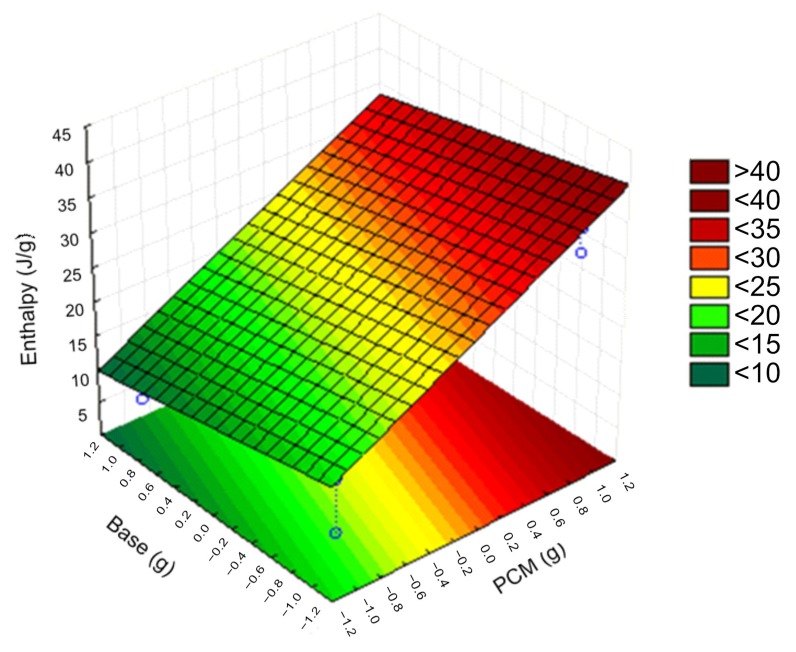
Response surface of the enthalpy to the most significant variables.

Figure 7 shows the 3D response surface with the amount of PCM and the base chosen as the most relevant factors. These values have been normalized. The response surface was a linear plane, from which it follows that the greater the amount of PCM, the greater the enthalpy, and the greater the base, the lower the enthalpy. At the bottom of the figure are the contours for constant enthalpy. This figure is two-dimensional way of explaining a 3D surface, appreciating that the warmer surfaces are concentrated in the corner (dark red), and were the result of combining the minimum base and the maximum amount of PCM. The greater the amount of PCM, the better the use of the characteristics offered by these materials, such as the absorption/cession of heat energy, whereas epoxy resins are good insulators, attenuating these properties and inversely influencing the enthalpy of the composite material.

**Figure 8:**
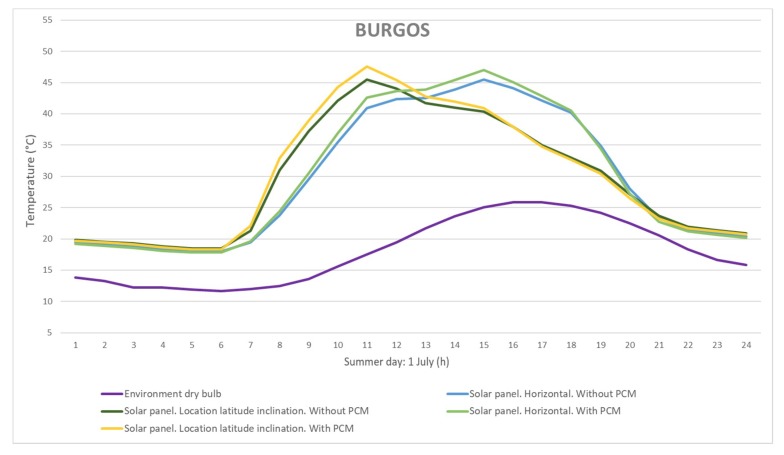
Estimated temperatures for a summer day in Burgos.

**Figure 9:**
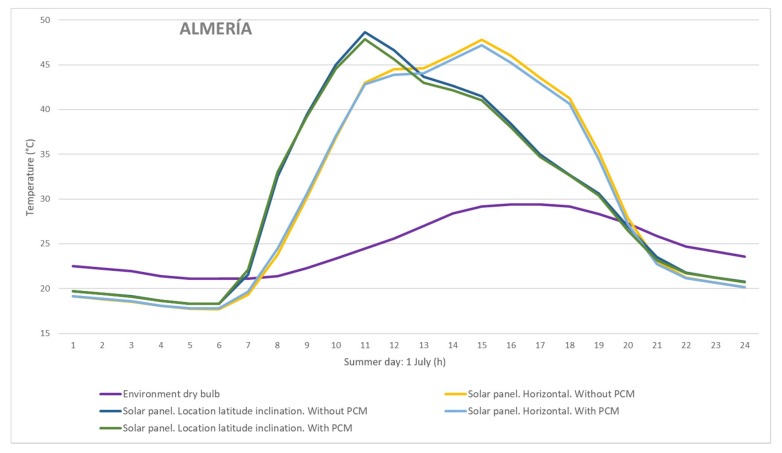
Estimated temperatures (°C) for a summer day in Almería.

The results showed that the inclusion of a PCM composite layer had an impact on solar panel temperatures, and therefore on panel performance. Although the selected climates were different, a decrease in panel temperature was observed in both cases. Burgos has a warm climate (Cfb Köppen–Geiger classification) and Almería has a steppe climate (BSk Köppen–Geiger classification). In the summer months, temperature drops of more than 4 °C were possible in Burgos, for the horizontal configuration. In Almería, it was possible to reduce the temperature of the panel by about 2 °C in the middle of the day during the summer period. As expected, higher temperatures were reached in the horizontal configuration. To show the effect of the PCM composite on the panel, the panel temperature is displayed during a summer day in the two locations (Figure 8 and Figure 9). The results achieved were due to the PCM transition temperature. Thus, depending on the climatic conditions of the location, the formulation of PCM can be more or less effective. This suggests that a specific formulation should be designed for each climatic zone. Moreover, the PCM layer added to the solar panel offered it high thermal inertia. This produced a delay and a reduction in the peak temperature.

## 4. Conclusions

In this work, we studied the behavior of an epoxy resin as a matrix to store phase change materials. A thickening agent and a material that increased thermal conduction were added to this mixture. The results showed how the resin acted as a matrix containing the PCM, preventing leakage in a liquid state and not affecting the heat storage capacity of the phase change material. Because encapsulation was not required, this is a more economical system.

Organic PCM turned out to be a reversible material in phase transformations when subjected to multiple cycles. Its temperature range of state change in the experimental tests was very similar to the theoretical one indicated by the manufacturer of the material. The amount of PCM added to the mixture had a direct influence on the thermal properties and an inverse influence on the mechanical properties. The addition of a thickening agent increased the homogeneity and compactness of the samples, allowing the manufacture of samples with about 40% PCM by weight without evident phase segregation. The addition of a metallic phase, aluminum in this study, to the resin–PCM composite produced an increase in heat flow for any amount of PCM in the sample. In addition, as was expected, only the PCM mass had a statistical impact on the enthalpy of the compound. However, aluminum tended to form non-metallic inclusions that impaired homogeneity. Therefore, it was concluded that the combination of resin, PCM, thickening agent, and conductive metal has a high heat absorption potential for application in surface coatings.

Lastly, using the PCM–epoxy composite for bottom-coating a photovoltaic coating could produce a cool down of several degrees Celsius compared to a panel without it. The improvement is better the warmer the climate of the photovoltaic facility. The estimation of the energy production improvements remains to be done in future works, as does the optimization of the PCM–epoxy for different weather conditions.

## Figures and Tables

**Table 1:** Plackett–Burman experimental design.

ID	A	B	C	D	E	F	G
**1**	−1	−1	−1	+1	−1	+1	+1
**2**	−1	+1	−1	−1	+1	−1	+1
**3**	−1	+1	+1	−1	−1	+1	−1
**4**	+1	+1	+1	+1	−1	−1	+1
**5**	−1	−1	+1	+1	+1	−1	−1
**6**	+1	+1	−1	+1	+1	+1	−1
**7**	+1	−1	+1	−1	+1	+1	+1
**8**	+1	−1	−1	−1	−1	−1	−1

**Table 2:** Values for the Plackett–Burman experiment.

Factors	Description	+1	%	−1	%
**A**	Resin Catalyst	8.52 g 2.98 g	42.60 14.90	12.37 g 4.33 g	61.90 21.60
**B**	Thickener	0.50 g	2.50	0.30 g	1.50
**C**	PCM	6.00 g	30.00	2.00 g	10.00
**D**	Metallic particles	2.00 g	10.00	1.00 g	5.00
**E**	Mixing time	30 min		10 min	
